# P-472. Development of a Local HIV Care Continuum for the Kansas City Metropolitan Area (KCMA)

**DOI:** 10.1093/ofid/ofae631.671

**Published:** 2025-01-29

**Authors:** Austin Price, Wissam El Atrouni

**Affiliations:** The University of Kansas Health System, Kansas City, Kansas; University of Kansas Medical Center, Kansas City, KS

## Abstract

**Background:**

There were 36,136 new HIV diagnoses in the USA in 2021. Still, an estimated 13% of persons living with HIV (PLHIV) are unaware of their infection. The status of the HIV epidemic can be better understood by constructing an HIV care continuum.

**Figure 1.**
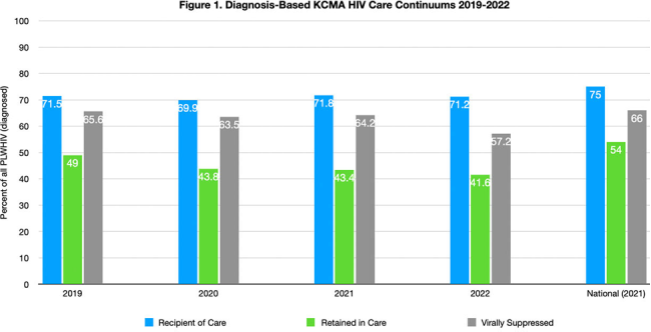
Diagnosis-Based KCMA HIV Care Continuums 2019-2022 Local diagnosis-based care continuums for 2019-2022 compared with the most recent national HIV surveillance data.

**Methods:**

HIV surveillance data reported to the Kansas Department of Health and Environment (KDHE) and Missouri Department of Health and Senior Services (DHSS) were utilized to create diagnosis-based HIV care continuums for 2019-2022. This included data from Johnson, Wyandotte, and Leavenworth counties in Kansas, and Jackson, Clay, and Platte counties in Missouri. Prevalence-based care continuums were constructed for Kansas City, Missouri for 2019-2021 utilizing the CD4-depletion model employed by CDC. Diagnosis, receipt of care, retention in care and viral suppression are per CDC definitions.

**Figure 2.**
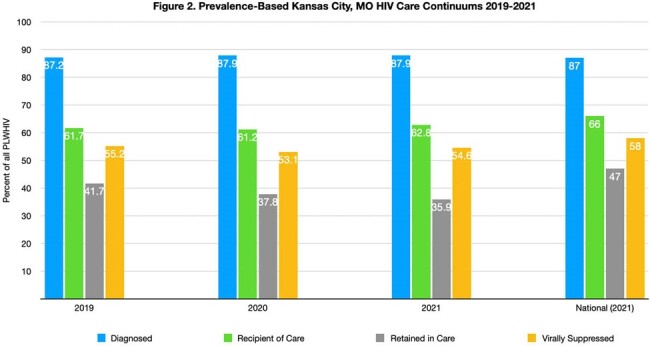
Prevalence-Based Kansas City, MO HIV Care Continuums 2019-2021 Local prevalence-based care continuums for 2019-2021 compared with the most recent national HIV surveillance data.

**Results:**

From 2019 to 2021, receipt of HIV care in KCMA remained stable, while retention in care (49% to 41.6%) and HIV viral suppression (65.6% to 57.2%) decreased (Figure 1). From 2019 to 2021, HIV diagnosis rates in the 3 counties of Kansas City, Missouri, remained stable at about 87%, but receipt of care rates decreased (41.7% to 35.9%), with stable viral suppression rates (∼54%) (Figure 2). Linkage to care improved from 70% to 75% for the 6-county KCMA from 2019 to 2022 (Figure 3).

**Figure 3.**
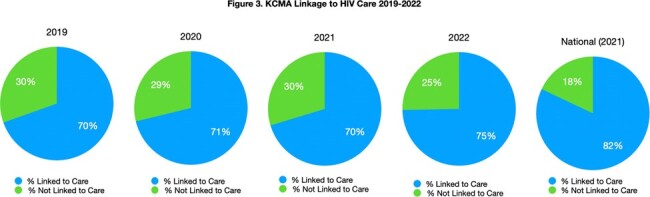
KCMA Linkage to HIV Care 2019-2022 Local linkage to HIV care estimates compared with the most recent national linkage to care estimates.

**Conclusion:**

Knowledge of status appeared to approximate national estimates. However, linkage to care was significantly lower (70-75% vs 82%), which likely negatively impacted downstream stages of the continuum. Local estimates for receipt of care, retention in care, and viral suppression were consistently below national levels. This highlights the importance of ongoing initiatives targeted at the more proximal stages. Reaching undiagnosed PLHIV requires a robust public health infrastructure which utilizes innovative diagnostic approaches to meet at-risk individuals in the spaces where they live and interact. Optimizing more distal stages of the continuum is contingent upon ongoing efforts to ensure all diagnosed PLHIV are efficiently linked to care and started on antiretroviral therapy (ART) in a timely fashion.

**Disclosures:**

**Wissam El Atrouni, MD**, ViiV Healthcare: Advisor/Consultant

